# Diet-Induced Alteration of Microbiota and Development of Obesity, Nonalcoholic Fatty Liver Disease, and Diabetes: Study Protocol of a Prospective Study

**DOI:** 10.2196/11553

**Published:** 2019-06-19

**Authors:** Martine Uittenbogaart, Wouter KG Leclercq, Danielle Bonouvrie, Marleen M Romeijn, Arijan APM Luijten, Steven WM Olde Damink, Francois MH van Dielen, Sander S Rensen

**Affiliations:** 1 Department of Bariatric Surgery Máxima Medical Center Veldhoven Netherlands; 2 Department of Surgery Maastricht University Medical Centre Maastricht University Maastricht Netherlands; 3 School of Nutrition and Translational Research in Metabolism Maastricht University Maastricht Netherlands

**Keywords:** microbiota, type 2 diabetes, obesity, NAFLD, gastric bypass

## Abstract

**Background:**

Development of obesity and obesity-related diseases, such as type 2 diabetes mellitus and nonalcoholic fatty liver disease (NAFLD), is associated with altered gut microbiota composition. The aim of this study is to investigate associations among dietary compounds, intestinal cell function, and gut microbiota composition. We hypothesize that dietary lipid intake is associated with Paneth cell and goblet cell properties that affect gut microbiota composition.

**Objective:**

The primary objective of this study is to determine whether a difference in dietary intake is associated with a difference in intestinal mucin-2 expression and gut microbiota composition.

**Methods:**

This is a single-center prospective study, including 1 obese group undergoing laparoscopic Roux-en-y gastric bypass and 2 lean control groups undergoing either laparoscopic cholecystectomy or upper gastrointestinal endoscopy (n=228). During laparoscopy, biopsies will be taken of visceral fat (omentum majus), liver, muscle tissue of the abdominal wall, and subcutaneous fat. In the obese group, a small segment of the jejunum will be collected for analysis, which will be compared with an endoscopically derived jejunal biopsy from the upper gastrointestinal endoscopy control group. Stool samples for microbiota profiling will be collected at baseline and 1 year after surgery. Primary outcomes are fecal microbiota composition and mucus characteristics. Secondary outcomes include Paneth cell phenotype, body weight, diet composition, glucose tolerance, resolution of comorbidities, and weight loss 1 year after surgery.

**Results:**

This trial is currently open for recruitment. The anticipated completion date is December 2019.

**Conclusions:**

The Diet-Induced Alteration of Microbiota and Development of Obesity, NAFLD, and Diabetes study will improve insight into the pathophysiology of obesity and its associated metabolic disorders. Better understanding of weight loss failure and weight regain following bariatric surgery might also behold new therapeutic opportunities for obesity and obesity-related comorbidities.

**Trial Registration:**

Netherlands Trial Register NTR5660; https://www.trialregister.nl/trial/5540 (Archived by WebCite at http://www.webcitation.org/78l7jOZre)

**International Registered Report Identifier (IRRID):**

DERR1-10.2196/11553

## Introduction

Obesity-associated diseases, such as type 2 diabetes mellitus (T2DM) and nonalcoholic fatty liver disease (NAFLD), are major public health issues worldwide, affecting more than 6% and 25% of the world population, respectively [1,2]. The influence of gut bacteria on the development of obesity and metabolic syndrome is not entirely understood.

Numerous experimental studies show that gut bacteria are influential in the development of obesity. For example, transplantation of gut microbiota from obese into germ-free mice has been shown to cause a higher fat mass increase than transplantation of lean microbiota. [3] Thus, altering gut bacterial composition can have a direct effect on body weight. In addition, gut microbiota might play a potential role in the treatment of T2DM. Indeed, targeting gut microbiota by antibiotic treatment has been shown to improve body weight and glucose tolerance of high-fat fed mice [4,5]. Prebiotic, as well as probiotic, treatment also improves glucose metabolism in high-fat diet-induced diabetes [6,7]. Moreover, infusion of gut microbiota from lean human donors into subjects with metabolic syndrome has been reported to result in increased insulin sensitivity [8], demonstrating the feasibility of gut microbiota modulation for improving glucose homeostasis in a clinical setting. NAFLD is the hepatic manifestation of the metabolic syndrome, characterized by ectopic fat accumulation in the liver. Previous animal studies have indicated a link between development of NAFLD and gut microbiota. For example, gut microbiota transplantation from mice with NAFLD to wild-type recipients led to replication of the NAFLD phenotype, showing that NAFLD is transmissible through gut bacteria [9]. The factors that underlie the microbiota alterations in obesity, T2DM, and NAFLD are unclear, although the genetic makeup of the host is considered to play a significant role. Gut microbiota composition and function are strongly affected by endogenous antimicrobial proteins secreted by Paneth cells in the small intestine [10], as well as by mucus components made by intestinal goblet cells [11]. Next to these host factors, dietary macronutrient composition has a strong impact on gut microbiota [12]. In particular, high-fat diets decrease abundance of *Akkermansia muciniphila*, a mucus associated bacterium that has been found to be inversely correlated with body mass in mice [13]. Besides this specific bacterium, diet also has an effect on the ratio of 2 major intestinal bacterial phyla: high-fat diets induce Firmicutes while reducing Bacteroidetes [3,14,15].

The data on changes in gut microbiota composition after bariatric surgery are relatively limited. Within 3 months after Roux-en-Y gastric bypass (RYGB), gut microbiota has been found to be more diverse with an increased relative abundance of *Akkermansia muciniphila* [16]. However, most of the available clinical studies have small sample sizes and only analyze fecal samples collected at 12 months postoperatively or less. The possible impact of gut microbiota on failure to maintain weight loss after bariatric surgery is still unknown. This phenomenon, better known as secondary nonresponse, can occur in up to 25% of all patients who undergo RYGB surgery, and it can become apparent at 12 to 24 months postoperatively [17,18]. A recent study showed no difference in microbiota composition between a group of patients following RYGB surgery who obtained more than 50% excess weight loss after 2 years and a group of patients who did not reach that weight goal [19]. Unfortunately, that study lacked baseline samples, and that study had a very small sample size. The overall aim of the proposed study is to investigate associations among intake of dietary compounds, intestinal cell function, and gut microbiota composition. The primary objective is to determine the relationship between diet and intestinal mucin-2 expression in obesity. Secondary objectives are assessment of the relationships among diet, intestinal goblet cell and Paneth cell function, and gut microbiota composition, as well as changes in these parameters in association with secondary nonresponse, presence of T2DM, and NAFLD. We hypothesize that dietary lipid intake is associated with Paneth cell and goblet cell properties that affect gut microbiota composition.

## Methods

### Study Design

This study will be conducted as a single-center prospective study, and it comprises a cross-sectional and a longitudinal part. In the cross-sectional part, differences at baseline between severely obese patients and lean subjects will be studied, focusing on the presence and severity of NAFLD, insulin resistance, T2DM and intestinal microbiota composition, Paneth cell products, and mucus composition. The longitudinal part will focus on changes in microbiota composition, Paneth cell products, and mucus in the severely obese group between baseline measurement and 1 year after RYGB.

### Ethical Approval and Recruitment

The Diet-Induced Alteration of Microbiota and Development of Obesity, Nonalcoholic Fatty Liver Disease, and Diabetes (DIAMOND) study is registered within the Netherlands National Trial Register (NTR560). The protocol was ethically approved by the official Independent Ethics Review Board of Máxima Medical Centre (reference 15.053) in November 2015. Written informed consent will be obtained from all participants. The study will be performed in accordance with the principles of the Declaration of Helsinki, as well as the guidelines of Good Clinical Practice. Recruitment started in the first quarter of 2016, and recruitment is currently ongoing. Patients deemed eligible for enrollment in either the obese group or the lean control (cholecystectomy) group are initially recruited by their surgeon or a specialized gastroenterology nurse at the time of approval for surgery or endoscopy. If interested in participation, the patient is contacted by the researcher and given detailed information about the study, in both oral and written form. After a 2-week period, the subjects are contacted to obtain informed consent, and then they will be officially enrolled in the study.

### Setting

Recruitment of patients and subsequent sampling are performed in a large teaching hospital in the Netherlands, which is awarded as a Center of Excellence in Metabolic and Bariatric Surgery.

### Study Population

The study population will comprise 3 groups: the obese group and 2 lean control groups. For the obese group, patients are screened by a multidisciplinary team, and they are approved for surgery according to the International Federation for the Surgery of Obesity and Metabolic Disorders guidelines. All patients with severe obesity and undergoing laparoscopic RYGB are considered eligible for inclusion. The surgical procedure is performed according to the circular stapling technique described by Dillemans et al [20].

Further inclusion criteria are a body mass index (BMI) between 35 and 45 kg/m^2^ and willingness to sign the informed consent form. Patients are excluded on the basis of the following criteria: (1) age<18 or >65, (2) presence of type 1 diabetes, substance abuse, inflammatory diseases, or neoplasms, (3) chronic use of corticosteroids prescribed by a physician, and (4) use of antibiotics in the 3 months preceding surgery.

**Figure 1:**
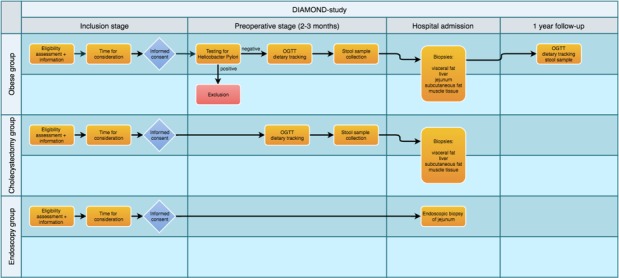
Flow chart. DIAMOND: Diet-Induced Alteration of Microbiota and Development of Obesity, Nonalcoholic Fatty Liver Disease, and Diabetes; OGTT: Oral Glucose Tolerance Test.

For the 2 lean control groups, all patients with a BMI between 20 and 25 kg/m^2^ undergoing either upper gastrointestinal endoscopy or a laparoscopic cholecystectomy are eligible for inclusion. Patients are excluded on the basis of the following criteria: (1) age<18 or >65, (2) presence of type 1 or T2DM, substance abuse, inflammatory diseases, or neoplasms, (3) chronic use of corticosteroids prescribed by a physician, (4) use of antibiotics in the 3 months preceding surgery or endoscopy, and (5) presence of cachexia, defined as unintended weight loss (>5% in 1 month or >10% in 6 months). A flowchart for each of the groups is provided in Figure 1.

### Data Collection

#### Characteristics

Phenotyping of obesity will be based on measurement of body weight and calculation of BMI. Presence and severity of T2DM will be assessed by analysis of hemoglobin A1_c_ in blood, as well as analysis of plasma glucose and insulin levels, both fasting and during an oral glucose tolerance test. Presence of NAFLD will be determined on the basis of the liver biopsy, according to the validated Kleiner score [21]. Plasma levels of aspartate transaminase and alanine transaminase will be measured as markers of liver damage. Furthermore, plasma lipid spectrum, total leukocyte count, and differentiation count, as well as C-reactive protein, will be measured.

#### Dietary Habits

Dietary habits will be recorded using a Dutch food tracker [22], which can either be used as a Web-based program or as a smartphone app. All participants will be asked to record their diet for the duration of 7 consecutive days at baseline. The obese group will repeat this at 1-year follow-up.

#### Intestinal Microbiota Composition

Participants will be provided a stool sample collection kit and will be asked to sample their stool before surgery. The samples will be stored in the home freezer (–20°C) and transported to the hospital on the day of admission, where they will be stored in the laboratory freezer (–80°C) until analysis. Participants in the obese group will be asked to provide a second stool sample at 1-year follow-up. Stool samples will be analyzed according to the shotgun metagenomics approach. By sequencing the whole genome, we will be able to compare this with a reference genome and evaluate abundance of DNA fragments. This will include detecting the 2 primary intestinal phyla—Firmicutes and Bacteroidetes—as well as *Akkermansia muciniphila* abundance. Furthermore, we will analyze the fecal samples for presence of short-chain fatty acids, that is, acetate, propionate, and butyrate.

#### Biopsies

In both the obese group and the lean control group, the following biopsies will be performed during laparoscopic surgery: visceral fat (omentum majus), liver, muscle tissue of the abdominal wall, and subcutaneous fat. In the obese group, a small segment of the jejunum will be collected during laparoscopic RYGB, a standard element of the procedure, and it will be used for analysis. In the control group, the jejunal full-thickness biopsy will be substituted by the endoscopically derived jejunal biopsy in the endoscopy group.

Directly after sampling, all tissues will be flash frozen and stored in the laboratory freezer (–80°C) until analysis. According to standard operating procedure protocol, the sampling in the obese group and lean cholecystectomy group will be performed by 3 surgeons involved in this study. The sampling in the lean endoscopy group will be performed by 1 gastroenterologist. The researcher will be present at the operating room and at the endoscopy suite at each procedure to flash freeze the samples directly after the biopsy. Stored data and materials will be only identifiable to the person by an assigned subject number. As such, patient privacy is guaranteed according to the Dutch Personal Data Protection Act.

The jejunal samples will be analyzed by means of quantitative PCR, specifically investigating HD5/DEFA5 lysozyme, mucin-1, and mucin-2, as well as Kruppel like factor 4 messenger RNA expression. Furthermore, a hematoxylin and eosin staining will be performed to allow for Paneth cell quantification. Liver samples will be taken to assess the presence and severity of NAFLD and nonalcoholic steatohepatitis, the use of the Kleiner score [21] will be done by a dedicated pathologist. Muscle tissue, visceral fat, and subcutaneous fat will be stored for future purposes to enable more detailed interorgan investigations of peripheral tissues involved in glucose and lipid homeostasis.

### Statistical Analysis

#### Power

Sample size is calculated on the basis of the smallest expected difference in the main outcome parameter, that is, intestinal mucin-2 expression, using G*power 3 (Erdfelder, Faul, and Buchner, 1996). A pilot study with lean nondiabetic and obese diabetic rats revealed a difference in expression of 1.00±0.71 versus 0.65±0.78. Taking this into account and using a significance level of 0.05 and a power of 80%, each group will require 73 patients. Considering an expected loss to follow-up of 5%, 76 patients need to be included in each group, amounting to 228 patients in total.

#### Analysis of Primary and Secondary Outcome Parameters

SPSS will be used for statistical analysis (IBM Corp, Released 2013, IBM SPSS Statistics for Macintosh, Version 22.0.). A 2-tailed *P* value <.05 will be considered statistically significant. To allow comparisons among groups, data will be tested for normal distribution, and appropriate statistical tests will be applied, potentially including Students *t* test, Mann-Whitney U test, analysis of variance, Kruskal-Wallis test, Chi-square test, or Fisher exact test.

### Ethics Approval

Ethics approval was granted by the Máxima Medical Centre Ethics Committee (reference 15.053) in November 2015.

## Results

This trial is currently open for recruitment. The anticipated completion date is December 2019.

## Discussion

The field of microbiota research is rapidly expanding and a plethora of links among diseases like obesity, T2DM or NAFLD, and gut microbiota composition are currently unraveled. However, a majority of novel findings in this context are based on animal models and remain to be substantiated in humans. The DIAMOND study aims to identify associations among dietary compounds, gut microbiota composition, and Paneth cell function, as well as intestinal mucus characteristics in man.

A limitation of the DIAMOND study design is the inability to prove causality. Cancelling out potential direct effects of dietary compounds on microbiota and metabolism, such as fermentation of dietary fibers to produce short-chain fatty acids [23], is unattainable. In addition, obtaining new biopsies after 1 year of weight loss to identify the impact of weight loss on primary and secondary outcomes is not implemented in this study, as it is considered too invasive.

In conclusion, the DIAMOND study will explore whether Paneth cell function and mucus composition are associated with diet and alterations in gut microbiota composition, and the study will investigate the impact of RYGB-induced weight loss on these parameters. This will not only benefit our understanding of weight loss failure and weight regain following bariatric surgery, but it might also behold new therapeutic opportunities for obesity and obesity-related comorbidities.

## Abbreviations

BMI: body mass index
DIAMOND: Diet-Induced Alteration of Microbiota and Development of Obesity, Nonalcoholic Fatty Liver Disease, and Diabetes
NAFLD: nonalcoholic fatty liver disease
RYGB: Roux-en-Y gastric bypass
T2DM: type 2 diabetes mellitus
